# Thermal near-field scattering characteristics for dielectric materials

**DOI:** 10.1038/s41598-023-44920-y

**Published:** 2023-10-16

**Authors:** Ryoko Sakuma, Kuan-Ting Lin, Yusuke Kajihara

**Affiliations:** 1 Department of Precision Engineering, The University of Tokyo, Bunkyo-ku, Tokyo, 113-8654 Japan; 2 Institute of Industrial Science, The University of Tokyo, Tokyo, Meguro-ku, 153-8505 Japan; 3 PRESTO, Japan Science and Technology Agency, Kawaguchi-shi, Saitama, 332-0012 Japan

**Keywords:** Optics and photonics, Optical physics

## Abstract

In this study, we passively analyzed the near-field characteristics of thermally excited evanescent waves, which are radiation waves generated by the local dynamics of materials, including electron motions and lattice vibrations. The thermally excited evanescent waves on aluminium nitride (AlN) and gallium nitride (GaN) were measured using passive spectroscopic scattering-type scanning near-field optical microscopy (s-SNOM) in the wavelength ranges of 10.5–12.2 μm and 14.0–15.0 μm, which include the surface phonon-polariton (SPhP) wavelength of the studied dielectrics. We determined the unique decay characteristics of AlN and GaN, indicating a ten-fold increase in the probe area contributing to the scattering of waves near the SPhP wavelength compared to that in other wavelength ranges. The extended probe area correlated with the polariton decay lengths, indicating that the non-enhanced polaritons around *K* ~ *ω*/c were dominant in the scattered waves near the SPhP wavelength. In addition to the conventional passive detection mechanisms for metals, the proposed detection scheme will be a versatile passive detection model in the near future.

## Introduction

All matter radiates heat unless it is at an absolute zero temperature. Randomly changing charge distributions owing to local fluctuations, including electron motions and lattice vibrations, can generate strong localized electromagnetic (EM) waves on a material surface—that is, thermally excited evanescent waves (wavelength range: 8–20 μm)^1^. By detecting these thermally excited evanescent waves, local information on the material’s dynamic properties can be obtained.

Scattering-type scanning near-field optical microscopy (s-SNOM) is one of the most widely used nanoscale measurement tools for analyzing local material properties. The scanning probe scatters localized waves below the probe apex and provides information on the near-field EM waves induced by localized physical or chemical phenomena^2^. In most conventional s-SNOM techniques, excited waves induced by external illuminations are detected to obtain a localized optical response^3–5^. s-SNOM with external illumination is called active-type s-SNOM and can be used to analyze excited waves—including light-plasmon coupling^6^, light-phonon coupling^7^, and molecular fluorescence resonance^8^. However, thermally excited evanescent waves cannot be detected using active-type s-SNOM because strong external illumination disturbs the local dynamics—that is, an s-SNOM technique without external illumination (passive type) is required to analyze local dynamics^9^.

Over the last decade, passive-type s-SNOM in the long-wavelength infrared (LWIR) range has been developed^10^. It comprises a charge-sensitive infrared phototransistor (CSIP)^11^, confocal optics, and a shear-force-based atomic force microscope (AFM). The noise equivalent power of the CSIP is ~ 7 × 10^−20^ W/Hz^1/2^^12^, making possible the detection of thermally excited evanescent waves without the use of external illumination. The passive s-SNOM technique has been applied to the nanoscale measurement of lattice (Joule heating) and electron (internal energy density) temperatures^13,14^. Moreover, a principle for the passive near-field detection of metals has been developed, and used to demonstrate that only the forefront of the probe apex was dominant in the scattering of evanescent waves^15^.

**Figure 1:**
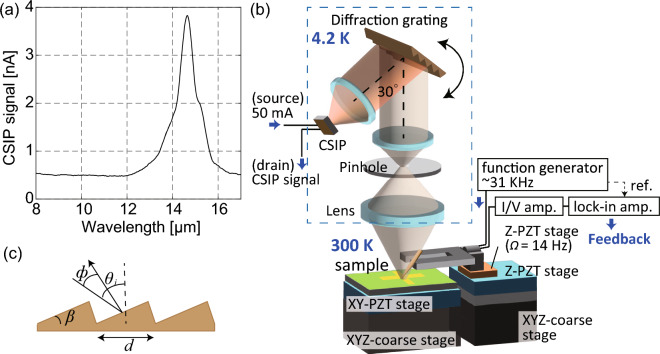
Passive LWIR spectroscopic s-SNOM system. (**a**) Optical response of the CSIP (detection wavelength: 14.5 μm). (**b**) Schematic of the passive LWIR spectroscopic s-SNOM. (**c**) Schematic representation of the blazed grating (*d* = 15.5 μm, *β* = 17°).

In the active s-SNOM measurements in the LWIR range, optical phonons and phonons are coupled in a specific condition, and the surface phonon polaritons (SPhP) emerge for many materials^6,7^. SPhPs are excited in the absorption band called the Reststrahlen band that is an energy band between the transverse-optic (TO) and longitudinal-optic (LO) phonon frequencies^16^. The Reststrahlen band exhibits high reflectivity as waves cannot propagate within the medium. The SPhPs have been analyzed using active-type s-SNOM; however, the localization characteristics of the thermally excited evanescent waves in the Reststrahlen band without light exposure have not been discussed owing to the lack of an ultra-sensitive passive detection technique. To clarify the passive detection characteristics, a thorough passive analysis near the surface phonon-polariton (SPhP) wavelength—that is a wavelength around *K* ~ $\omega /c$ where polaritons emerge—is required. Note that the SPhPs are ultra-weak in the passive detection. To analyze the detection characteristics of thermally excited evanescent waves—especially near the SPhP wavelength—a wavelength resolution of 100–200 nm is required as the energy density around the SPhP wavelength changes considerably. However, the detected signal using passive-type s-SNOM is an integral of the CSIP optical response, the wavelength resolution being ~ 1–2 μm. The CSIP optical response is as shown in Fig. 1a. In this study, we used a passive LWIR spectroscopic s-SNOM device, fabricated using a grating-based spectroscopic mechanism, with a wavelength resolution of ~ 150 nm^17^. We performed passive near-field measurements of aluminum nitride (AlN) and gallium nitride (GaN), in which their SPhP wavelengths were 11.8 and 14.1 μm, respectively. We compared the characteristics of the passively detected near-field signals of Au, AlN, and GaN and examined the differences in the scattering mechanism of the evanescent waves.

The principal aim of this study is to clarify the characteristics of thermally excited evanescent waves of dielectric materials in the Reststrahlen band and develop a principle for the passive near-field detection of dielectric materials. We performed a near-field decay analysis—measuring changes in the near-field signal using different probe heights—and calculated the probe area that contributed to the scattering of the evanescent waves. We demonstrated that the polarized area of the probe apex contributing to wave scattering extended only in the Reststrahlen band. The tip-polarized area in the Reststrahlen band had correlation with the polariton decay length, in which local minimum was found at the SPhP wavelength. The extended polarized area demonstrated that the characteristics of the detected near-field signals were determined primarily by waves with lower fluctuation modes, that is non-enhanced polaritons around *K* ~ $\omega /c$.

## Result

### The passive spectroscopic s-SNOM mechanism

Figure 1b shows a schematic of the passive LWIR spectroscopic s-SNOM mechanism^17^. The thermally excited evanescent waves are scattered using the AFM scanning probe, and the scattered waves are collected by means of a Ge objective lens. The waves collected by the Ge objective lens pass through a pinhole of diameter 250 μm; waves outside of the focus position are removed. The spatial resolution is 50–150 nm, depending on the apex radius of the scanning probe. A specific wavelength can be selected by rotating the diffraction grating using a piezoelectric stage. Waves reflected at 30° from the incident light are detected at the CSIP. The diffraction grating is a blazed-type grating with a rectangular apex—the grating pitch and the blazed angle being 15.5 μm and 17°, respectively—as shown in Fig. 1c. A blazed-type grating is used in spectroscopic optics because it has a higher diffraction efficiency than other grating structures^18^. The first-order diffraction light being detected primarily at the CSIP^19^.

**Figure 2:**
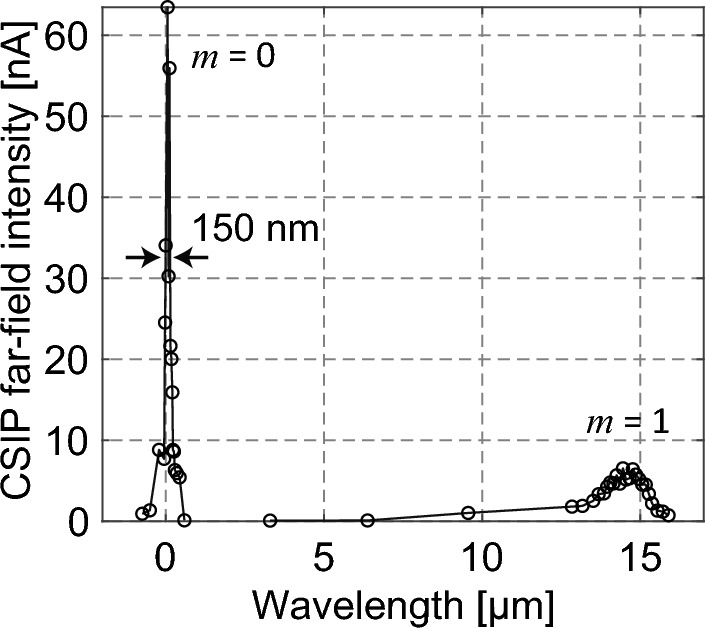
Far-field spectrum of a 500 K heat source obtained using the 14.5-μm CSIP.

With passive LWIR spectroscopic s-SNOM, two different diffraction lights can be detected—namely, the zeroth- and first-order diffracted light. The detection wavelength can be calculated using the grating equation, $m\lambda =d(\mathrm{sin}{\theta }_{i}+\mathrm{sin}({\theta }_{i}-\phi ))$, where *m*, *λ*, *θ*_*i*_, and $\phi$ (30°) denote the diffraction order, detection wavelength, incident angle to the normal of the grating, and angle between the incident and reflected light, respectively^20^. Figure 2 shows the far-field spectrum of a heat source (temperature: ~ 500 K) obtained using a CSIP of detection wavelength 14.5 μm (14.5-μm CSIP) without using a scanning probe. Here, radiative heat—in which the energy density is determined by Planck’s law—is detected. The actual rotation angle of the diffraction grating is measured using a capacitance encoder^17^. A strong signal owing to the zeroth-order diffracted light (*m* = 0) is evident at *λ* = 0 μm. The FWHM of the zeroth-order peak is ~ 150 nm, indicative of the wavelength resolution of the passive LWIR spectroscopic s-SNOM mechanism. A smaller peak in the wavelength range of 13.8–15.2 μm is a detection peak of the first-order diffracted light (*m* = 1), the detection range depending on the optical response of the CSIP.

### Probe-scattered evanescent waves

The total energy of the thermally excited evanescent waves can be expressed as a product of an EM local density of states (EM-LDOS: $\rho \left(z, \omega \right)$) and Bose–Einstein distribution $u\left(z, \omega , T\right)=\rho \left(z, \omega \right)\hslash \omega /(\mathrm{exp}\left(\hslash \omega /{k}_{B}T\right)-1)$, where *z*, *ω*, ${k}_{B}$, and* T* denote the sample-probe distance (Fig. 4b), angular frequency, Boltzmann constant, and medium temperature^1^, respectively. The energy intensity of the thermally excited evanescent waves generated at room temperature is proportional to the EM-LDOS of the sample. The EM-LDOS at T = 300 K can be expressed as follows^21^:

1 $$\begin{aligned} \rho \left( {z, \omega } \right) = & \frac{{\omega^{2} }}{{2\pi^{2} c^{3} }}\left\{ {\mathop \int \limits_{0}^{1} \frac{\kappa d\kappa }{p}\left\{ {2 + \kappa^{2} \left[ {{\text{Re}}\left( {r_{12}^{s} e^{2ip\omega z/c} } \right) + {\text{Re}}\left( {r_{12}^{p} e^{2ip\omega z/c} } \right)} \right]} \right\}} \right. \\ & \quad + \left. {\mathop \int \limits_{1}^{\infty } \frac{{\kappa^{3} d\kappa }}{\left| p \right|}\left[ {{\text{Im}}\left( {r_{12}^{s} } \right) + {\text{Im}}\left( {r_{12}^{p} } \right)} \right]e^{ - 2\left| p \right|\omega z/c} } \right\} \\ \end{aligned}$$

where ${r}_{12}^{s}$, ${r}_{12}^{p}$, $c$ denote the Fresnel reflection factors in *s* and *p* polarizations, the speed of light in a vacuum. The lateral and vertical wave vectors are presented with normalized vectors $\kappa$ and $p$, where $\kappa =Kc/\omega$ and $p=\sqrt{1-{\kappa }^{2}}$ if $\kappa <1$ and $p=i\sqrt{{\kappa }^{2}-1}$ if $\kappa >1$. Here, ${\varepsilon }_{1}$ and ${\mu }_{1}$ denote dielectric and magnetic constants. The integrals from $\kappa =0$ to 1 and $\kappa =$ 1 to $\infty$ reveal the EM-LDOS of propagating and evanescent waves^21^. Electromagnetic waves with $\kappa \sim 1$ are named polariton components in this paper, which are often sensed in the active s-SNOM measurement (with external illumination) after strong enhancement. Again, note that the resonance states (polaritons) are weak in the passive measurements.

**Figure 3:**
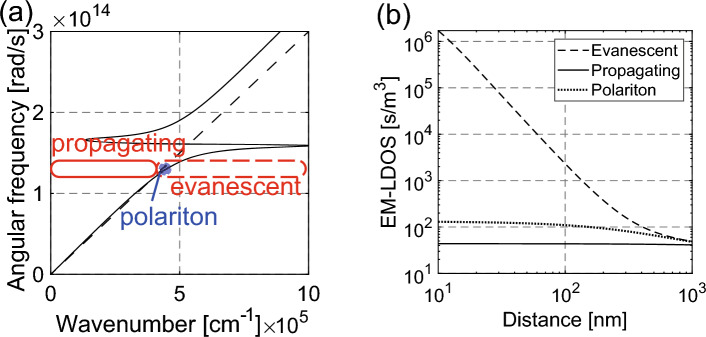
Characteristics of the thermally excited evanescent waves. (**a**) Dispersion relationship of AlN (solid line) and the light line (dashed line). The propagating, evanescent, and polariton components are indicated by the solid, dashed, and dotted lines, respectively. (**b**) Decay characteristics of the EM-LDOS of AlN at a wavelength of 14.5 μm.

Figure 3a shows the dispersion relationship of AlN (solid line) with the light line (dashed line) calculated using the Lorentz model. The parameters used for the calculation can be found in Supplementary Materials. The propagating and evanescent components are indicated by the solid and dashed red lines, respectively. The polariton component—which is often detected with active-type s-SNOM—is indicated by the filled blue area. Figure 3b shows the change in the EM-LDOS of the thermally excited evanescent waves with distance from the AlN surface at a wavelength of 14.5 μm^17^. The evanescent component is 10^2^–10^4^ times stronger than the propagation waves within 100 nm of the material surface and decays exponentially.

During near-field measurements, the CSIP senses signals owing to background radiation in addition to near-field signals. To extract the near-field signal, the CSIP signal can be modulated by vertically vibrating the scanning probe on the sample. The probe vibration frequency (*Ω*) was 14 Hz and the amplitude 100–900 nm, depending on the sample material.

**Figure 4:**
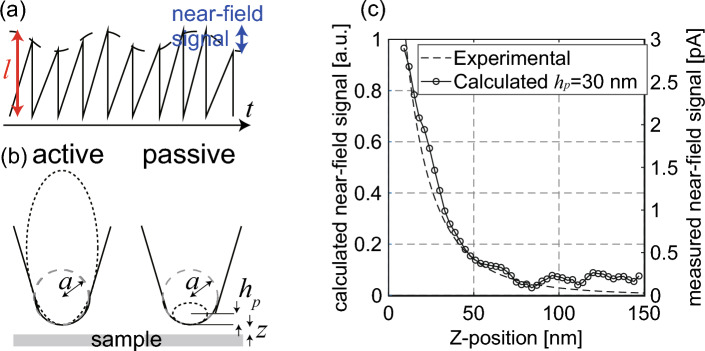
Near-field signals obtained with passive LWIR s-SNOM. (**a**) A schematic of the CSIP signal during the near-field measurements. (**b**) The probe-apex area contributing to the scattering of the evanescent waves in active and passive s-SNOM. (**c**) Calculated (*h*_*p*_ = 30 nm,* d* = 100 nm) and experimentally obtained near-field decay curve on Au at the fundamental frequency (*Ω* = 14 Hz) using passive LWIR spectroscopic s-SNOM (*m* = 0).

Figure 4a shows a schematic of the CSIP signal during the near-field measurements. The CSIP yields a sawtooth-shaped signal, the amplitude of which can be regarded as the CSIP signal intensity (*l* in Fig. 4a)^10^. The CSIP signal intensity increases when the probe apex is near the sample surface because the probe scatters thermally excited evanescent waves localized on the material surface. The change in the CSIP signal can be obtained as the near-field signal by using a lock-in amplifier with a reference to the probe-modulation frequency (*Ω* = 14 Hz). During passive near-field measurements on a metal in which external illumination is not used, only the forefront of the scanning probe contributes to the scattering of the evanescent waves because the scattered waves are primarily due to the evanescent field on the material surface, as shown in Fig. 4b^15^. However, in conventional active near-field measurements with external illumination, a larger area of the probe apex contributes to the scattering of EM waves because the external illumination generates a larger spot size, including the probe shaft (Fig. 4b)^22^. Consequently, the difference in the probe-apex area contributing to signal enhancement causes different decay characteristics of near-field signals^9^. In passive measurements, the decay length—which is the probe-sample distance when the intensity decreases to 1/*e*—is ~ 30 nm with a demodulating frequency of *Ω* (the fundamental frequency)^15^. Conversely, the decay length of the near-field signals obtained with active s-SNOM (with external illumination) is larger than 1 μm with a modulating frequency of *Ω* and it decreases to several tens of nanometers with higher harmonics (2*Ω* or 3*Ω*)^23,24^. The shorter decay length (< 100 nm) with *Ω* proves that the near-field signal does not include signals due to background radiation.

The near-field signals obtained at the CSIP are the interaction waves between the probe and the thermally excited evanescent waves. The probe apex is polarized by thermally excited evanescent waves, the probe-apex polarization enhancing the local EM waves. The tip-scattered near-field signals can be described using the Mie theory as follows^23^:

2 $$\begin{array}{*{20}c} {I \propto \left| {\alpha_{eff} \left( {z + h_{p} , \omega } \right)} \right|^{2} \rho \left( {z + h_{p} , \omega } \right) - \left| {\alpha_{eff} \left( {z + h_{p} + d, \omega } \right)} \right|^{2} \rho \left( {z + h_{p} + d, \omega } \right).} \\ \end{array}$$

where $d$ and ${h}_{p}$ denote the modulation amplitude of the scanning probe and the distance from the probe end to the center of the polarized area, respectively. Also, ${\alpha }_{eff}\left(z, \omega \right)$ denotes the effective polarizability ${\alpha }_{eff}=\alpha \left(1+\beta \right)/\left[1-\alpha \beta /16\pi {z}^{3}\right]$, where $\alpha =4\uppi {r}^{3}\left({\varepsilon }_{p}-1\right)/\left({\varepsilon }_{p}+1\right)$ and $\beta =\left({\varepsilon }_{s}-1\right)/\left({\varepsilon }_{s}+1\right)$. Here, ${\varepsilon }_{p}$, ${\varepsilon }_{s}$, and *r* denote the dielectric constant of the probe and sample materials and the effective radius of the probe apex, respectively. In Eq. (2), the temperature parameter is not included because all measurements were conducted at the constant temperature (T = 300 K). The signal intensity was only determined by the effective polarizability and EM-LDOS^9^. In most passive near-field measurements, the polarized area of the probe apex can be considered to be a spheroid with a vertical axis (*h*_*p*_), as shown in Fig. 4b^15^. Therefore, the effective radius (*r*) of the probe apex can be calculated using the actual tip radius (*a*)—that is, $r=\sqrt{\left({a}^{2}-{\left(a-{h}_{p}\right)}^{2}\right)}$.

In the near-field measurement, the detectable wavenumber is restricted by the radius of the probe apex—that is, $\omega /c<K<(\lambda /r)\times (\omega /c)$— because fluctuation modes with wavelengths smaller than the size of the probe apex cannot be scattered^15^. The polarized dipoles induced with different phases are cancelled out in the probe apex if the wavelength is smaller than the apex size. Figure 4c shows an example of the passively obtained near-field decay curve—reflecting the change in the near-field signal intensity with increasing sample-probe height—on Au using the 14.5-μm CSIP (*m* = 0) and calculated near-field signals using Eq. (2). The modulation amplitude ($d$) is 100 nm. The vertical axis of the polarized sphere (*h*_*p*_) can be determined by curve fitting using the near-field decay curve—that is, ${h}_{p}$ = 30 nm from the decay curve shown in Fig. 4c.

### Passive decay curves on dielectric materials

**Figure 5:**
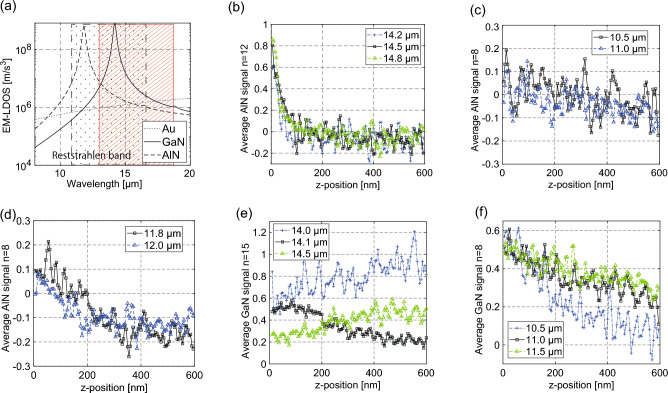
Near-field signals near the SPhP wavelength of AlN and GaN. (**a**) Calculated near-field signal intensities (*a* = 100 nm, *h*_*p*_ = 30 nm) of AlN (solid), GaN (dashed), and Au (dotted) at *z* = 10 nm. (**b**–**d**) Normalized near-field decay curves of AlN at a wavelength of (**b**) 14.2, 14.5, and 14.8 μm, (**c**) 10.5 and 11.0 μm, (**d**) 11.8 and 12.0 μm. The scattered points are the experimentally obtained near-field signals normalized to that of Au. (**e**), (**f**) Normalized near-field decay curves of GaN at a wavelength of (**e**) 14.0, 14.1, and 14.5 μm, and (**f**) 10.5, 11.0, 11.5 μm.

In the passive near-field measurement of metal, the intensity of the near-field signal decreases exponentially, as shown in Fig. 4c. Conversely, dielectric materials—including AlN and GaN—have their transverse-optic (TO) and longitudinal-optic (LO) phonon frequencies (${\omega }_{TO}$ and ${\omega }_{LO}$) in the LWIR range and can be expected to have unique detection phenomena. The spectral range between ${\omega }_{TO}$ and ${\omega }_{LO}$ is known as the Reststrahlen band^25^. The ${\omega }_{TO}$ and ${\omega }_{LO}$ are 610 cm^−1^ and 891 cm^−1^ for AlN, and 532 cm^−1^ and 734 cm^−1^ for GaN, respectively^26,27^. The Reststrahlen bands of AlN and GaN are 11.2–16.4 μm and 13.6–18.8 μm, as shown by the dot- and line-filled area in Fig. 5a, respectively. The solid, dashed, and dotted lines in Fig. 5a show the calculated EM-LDOS of AlN, GaN, and Au over a wavelength range of 8–20 μm at *z* = 10 nm. AlN and GaN exhibit their maximum EM-LDOS at the SPhP wavelengths which are 11.8 μm and 14.1 μm, respectively, these wavelengths (frequencies) being located between ${\omega }_{TO}$ and ${\omega }_{LO}$.

In this study, we used two CSIPs capable of different color detection—that is, the 14.5-μm CSIP (detection wavelength range: 13.8–15.2 μm) and the 11.3-μm CSIP (detection wavelength range: 10.5–12.2 μm)^12^. By using these two CSIPs, we could clarify the characteristics of the passively detected near-field signals near the SPhP wavelength of AlN (11.8 μm) and GaN (14.1 μm). We obtained the AlN and GaN decay curves and clarified the differences in the decay characteristics at each wavelength. As shown in Fig. 2, the intensity of the CSIP signal depends on the detection wavelength. Furthermore, the signal intensity changes depending on the size of the probe apex. The EM-LDOS of Au in the LWIR range is almost constant, as shown in Fig. 5a. Consequently, the intensity of the near-field signals on Au could be used as a reference to normalize the near-field signals of AlN and GaN to remove these influences. The SNR of the passive spectroscopic measurements were lower than that of the 0th order diffraction light, shown in Fig. 4c. The SNR obtained in measurements were less than 6.5 dB. The SNR of each measurement can be found in Supplementary Materials. All decay curves with uncertainties are also in Supplementary Materials.

Figure 5b shows the experimentally obtained near-field AlN decay curves (*d* = 100 nm) at wavelengths of 14.2, 14.5, and 14.8 μm. The radius of the probe’s apex (*a*) used for measurements was ~ 100 nm. These wavelengths are longer than the SPhP wavelength but still within the Reststrahlen band. The near-field signals decrease exponentially with a decay length of ~ 50 nm. The decay characteristics are similar to those of Au, as shown in Fig. 4c. The signal intensities near the surface are approximately 0.8 of those on Au and are qualitatively similar to the calculated EM-LDOS ratio, as shown in Fig. 5b.

Figures 5c,d show the near-field decay curves at $\uplambda <{\uplambda }_{\mathrm{SPhP}}$ (10.5, 11.0 μm) and $\approx {\uplambda }_{\mathrm{SPhP}}$ (11.8, 12.0 μm), respectively. When the detected wavelength is shorter than the SPhP wavelength, the decay lengths are ~ 200 nm, which are approximately five times longer than that measured at ~ 14.5 μm, the signal intensities being ~ 1/10 of those of Au. More unique decay characteristics are evident near the SPhP wavelength. The highest signal intensity is obtained at *z* =  ~ 50 nm, which gradually decays with a decay length of 180 nm.

The highest SNR in a range of $\uplambda \lesssim {\uplambda }_{\mathrm{SPhP}}$ is obtained at the SPhP wavelength (11.8 μm); however, the near-field signal intensity of AlN with regards to Au is extremely small compared to that of the calculated EM-LDOS ratio. Considering the extended decay lengths, all decay curves were obtained using a probe-modulation amplitude (*d*) of 330 nm.

Similar decay characteristics are evident in the near-field measurement of GaN, in which the polariton wavelength is at 14.1 μm. Figures 5e,f show the decay curves of GaN at $\sim {\uplambda }_{\mathrm{SPhP}}$ (14.0, 14.1, and 14.5 μm) and $\uplambda <{\uplambda }_{\mathrm{SPhP}}$ (10.5, 11.0, and 11.5 μm), respectively. The unique decay characteristics near the SPhP wavelength are more evident in the GaN measurements. The decay phenomenon is evident only at the SPhP wavelength (14.1 μm), the highest signal intensity being obtained at *z* = 120 nm and the decay length being ~ 250 nm (Fig. 5e). It should be noted that the decay curves shown in Fig. 5e were obtained using Rθ-lock-in detection—that is, the absolute signal intensity was measured where others were obtained using XY-lock-in detection.

The reasons for these unique decay characteristics near the SPhP wavelength are discussed in the following section. When the detected wavelengths are shorter than the SPhP wavelength, the near-field signals slowly decrease with a decay length of 150–250 nm (Fig. 5f). With XY lock-in detection, negative near-field signal intensities are obtained when *z* > 100 nm for AlN and *z* > 400 nm for GaN. The near-field signal can be obtained using a lock-in amplifier with reference to the tip-modulation vibration frequency; the negative intensity indicates that the phase was 180° with respect to the reference signal, the 180° phase change being caused by the detection of background radiation initially blocked by the probe shaft.

The near-field signal intensities with regard to the Au signal are qualitatively similar to the calculated EM-LDOS ratios when the detection wavelengths are several micrometers away from the SPhP wavelength. However, the signal intensities near the SPhP wavelength are lower than those of Au, differing considerably from the calculated EM-LDOS. The experimental results show that the characteristics of the detected near-field signals near the SPhP wavelength must be numerically described using a novel detection scheme.

### Discussion: the passive detection mechanism

The near-field decay curves of AlN and GaN at the SPhP wavelength show that the decay length is greatly extended, the signal strength being 10^2^–10^3^ times smaller than that of the calculated AlN/Au or GaN/Au EM-LDOS ratios. To describe these characteristics numerically, we assumed the probe apex to be a polarized sphere and proposed a novel detection scheme for passive near-field detection in the Reststrahlen band. The vertical axis of the polarized sphere (*h*_*p*_), as shown in Fig. 4b can be calculated by curve fitting. Conventionally, the effective radius of the probe apex is calculated as $r=\sqrt{\left({a}^{2}-{\left(a-{h}_{p}\right)}^{2}\right)}$; however, this can only be applied if *h*_*p*_ is sufficiently shorter than the actual tip radius (*a*)^15^. The longer decay length observed near the SPhP wavelength indicates that the polarized sphere in the probe apex is potentially larger than that used in the conventional model. Here, we assume $a\approx {h}_{p}$ or $a<{h}_{p}$, especially near the SPhP wavelength, because the decay lengths of the detected near-field signals on AlN and GaN are 100–250 nm, these being close to the radius of the probe apex. The ${h}_{p}$ of the polarized sphere in the conventional model was the minor axis; however, the ${h}_{p}$ of the detection model for the Reststrahlen band tends to be the major axis of the polarized sphere, similar to active s-SNOM.

**Figure 6:**
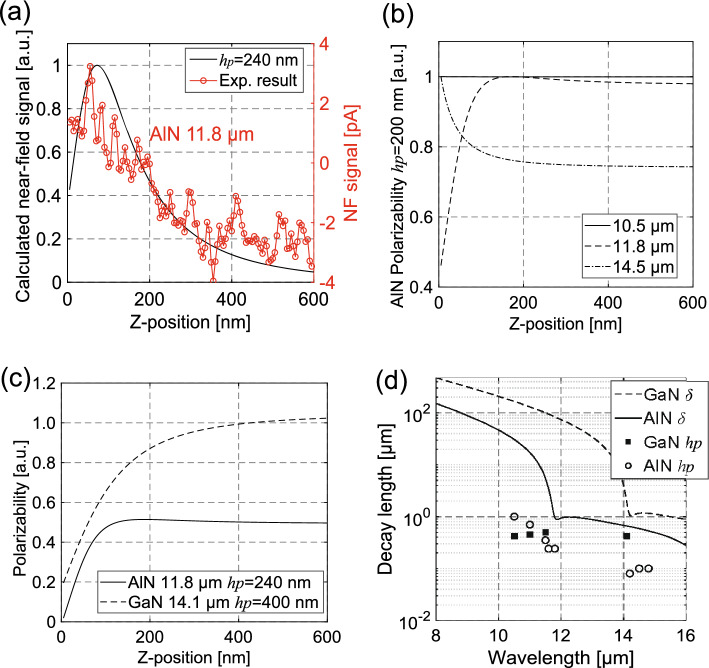
Result of the theoretical calculations using the proposed passive detection scheme. (**a**) Example of the curve fitting of AlN at 11.8 μm. (**b**) Change in the effective polarizability with distance from an AlN surface at wavelengths of 10.5, 11.8, and 14.5 μm. (**c**) Change in the effective polarizability of the W-AlN (wavelength: 11.8 μm) and W-GaN probing system (wavelength: 14.1 μm). (**d**) Experimentally obtained vertical axis of the polarized sphere *h*_*p*_ and polariton decay length of AlN (open circle, solid line) and GaN (filled square, dashed line).

We can describe the effective radius (*r*) by taking the geometric average of *a* and* h*_*p*_ ($r=\sqrt{a{h}_{p}}$). The vertical axis of the polarized sphere (*h*_*p*_) can be calculated via curve-fitting analysis using the experimentally obtained near-field decay curves at each wavelength. An example of the curve fitting of the AlN decay curve at a wavelength of 11.8 μm is shown in Fig. 6a. As expressed in Eq. (2), the near-field signal intensity can be described as the product of the EM-LDOS and the square of the effective polarizability. Therefore, the influence of effective polarizability on the detected near-field signal is substantial in s-SNOM measurements.

Figure 6b shows the calculated value of the normalized effective polarizability with increasing probe-sample distance at wavelengths of 10.5, 11.8, and 14.5 μm, assuming the probe and sample materials to be W and AlN, respectively. The *h*_*p*_ used in the calculations was 200 nm. The effective polarizability changes with distance and its characteristics vary considerably with wavelength—for example, when ${\lambda >\lambda }_{SPhP}$, the effective polarizability decreases exponentially; unique characteristics are evident at ${\lambda \lesssim \lambda }_{SPhP}$; it is almost unchanged at ${\lambda <\lambda }_{SPhP}$; and is at its maximum at *z* = 150 nm at ${\lambda \approx \lambda }_{SPhP}$. These results explain why the decay length is longer at ${\lambda <\lambda }_{SPhP}$ (Fig. 5c,f) and has its maximum at z = 50–120 nm near the SPhP wavelength (Fig. 5d,e). Similar characteristics are evident for the effective polarizability of the W–GaN probing system. The *h*_*p*_ on AlN at 11.8 μm and GaN at 14.1 μm, calculated using the decay curves shown in Fig. 5d,e, are 240 nm and 400 nm, respectively. Thus, the effective polarizability of the W-GaN probing system is twice that of the W-AlN probing system, as shown in Fig. 6c, which explains why the signal on GaN is stronger and clearer than that on AlN at the SPhP wavelength. The extended polarized area makes it difficult for the probe to scatter localized waves near the surface (z < 10 nm); thus, the intensity of the near-field signal near the SPhP wavelength decreases. The calculated *h*_*p*_ from the AlN and GaN decay curves at all detected wavelengths are shown as open circles and filled square plots in Fig. 6d. The solid and dashed lines show the polariton decay length of AlN and GaN, respectively. The ${h}_{p}$ near the SPhP wavelengths is approximately ten-fold larger than the *h*_*p*_ used in the conventional model for the passive near-field measurements^15^.

The calculated polarized areas of the probe apex in the W-AlN and W-GaN probing systems in the Reststrahlen band exhibit the following characteristics (plots in Fig. 6d)—that is, a long *h*_*p*_ (500–1000 nm) at ${\lambda <\lambda }_{SPhP}$, local minimum *h*_*p*_ (200–400 nm) at ${\lambda \approx \lambda }_{SPhP}$, and short *h*_*p*_ (80–100 nm) at ${\lambda >\lambda }_{SPhP}$. Similar decay characteristics appear in the polariton decay length ($\delta$)^1^—which is given by $\delta =1/\mathrm{Im}\left({\gamma }_{1}\right),{\gamma }_{1}^{2}={\varepsilon }_{1}{\mu }_{1}{k}_{0}^{2}-{K}^{2}$—as shown by the solid (AlN) and dashed (GaN) line in Figs. 6d and S2 (Supplementary Materials). The polariton decay length ($\delta$) is the penetration length of light in medium and only reflects the information of the polariton component ($K\sim \omega /c$). The minimum polariton decay length in the Reststrahlen band is found at the SPhP wavelength. The correlation between the polarized area of the probe apex and polariton decay length would be explained as follows. In this study, we confirmed that the polariton component is dominant in the Reststrahlen band. Therefore, the localization characteristics are mostly determined by that of the polariton components. The decay length of the evanescent waves in the Reststrahelen band is much longer than that out of the Reststrahelen band (> 1 μm) because of the coherence of the polaritons. It is obvious from calculations of the polariton decay length (Fig. S2 in Supplementary Materials). Therefore, the extended polarized area of the probe apex is owing to the long coherence of the polaritons, and the polarized area is correlated to the coherence length of the polaritons.

In principle, the passively obtained near-field signals are the integral of the wavenumbers in the range $\omega /c<K<(\lambda /r)\times (\omega /c)$, as expressed in Eq. (1). The scattered wavenumber range is restricted by the effective radius of the probe apex ($r$), with waves of higher fluctuation modes not being scattered. In the conventional passive detection model for metals, the *h*_*p*_ is ~ 30 nm, and thus the scattered near-field signals include higher-fluctuation modes. However, in the near-field measurement of dielectric materials—especially in the Reststrahlen band—the polarized area is an order of magnitude larger than that in the conventional model and thus the scattering characteristics differ from those outside the Reststrahlen band.

In the proposed detection scheme, we assume that waves with higher-fluctuation modes are difficult to scatter and are not included in the calculation of the tip-scattered near-field signals. However, there is still a possibility that higher-fluctuation modes are detectable with a smaller probe apex polarized area, and super-small signals owing to the higher-fluctuation modes can be detected. The results show that the polariton components are dominant in the near-field signals in the Reststrahlen band, but this does not mean that the higher-fluctuation modes have been completely canceled.

In this study, we clarify the correlation between the polarized area of the probe apex and the polariton decay length in the Reststrahlen band. A novel passive detection scheme that considers an extended polarized area is the basis for further theoretical development. To clarify the characteristics in the passive near-field measurement, we conducted passive decay analysis of AlN and GaN in distinct wavelength ranges (10.5–12.2 μm and 14.2–14.8 μm, respectively) because the detection wavelengths are limited by the optical property of the CSIP. A more detailed analysis would be possible using a CSIP with an extended detection range. Recently, a multicolor CSIP with a two- or three-fold detection wavelength range was developed^28^. Multicolor CSIPs provide continuous spectral information, which can help to clarify the passive detection mechanism more accurately.

## Conclusion

In this study, we conducted a passive decay analysis of thermally excited evanescent waves using passive LWIR spectroscopic s-SNOM. The unique near-field decay characteristics of AlN and GaN were observed near the SPhP wavelengths and were attributed to the extended vertical axis of the polarized sphere in the probe apex, which contributed to the scattering of thermally excited evanescent waves. In the conventional passive detection model applied to the measurement of a metal surface, it is assumed that only the forefront of the probe tip contributes to the scattering of evanescent waves; thus, fluctuation modes with higher wavenumbers are included in the scattered waves. In this study, we proposed a passive detection scheme near the SPhP wavelength with an extended polarized area. The vertical axis of the polarized sphere in the Reststrahlen band correlated with the polariton decay length, indicating that the polariton components with lower fluctuation modes were more dominant in the scattered near-field signals. This finding provides a basic model for detecting dielectric materials. In addition to the conventional passive detection mechanisms for metals, a more versatile detection model will be developed in the near future. Passive LWIR spectroscopic s-SNOM with a versatile detection model will be a powerful tool for analyzing the local dynamics.

## Method

### Probe fabrication

The scanning probe was fabricated by electrochemical etching of a tungsten (W) wire of diameter 50 μm. The tungsten wire was dipped into the electrolyte (33% KOH solution) and 950 mV AC voltage was applied. The etching was automatically stopped when the current reached 0.02 mA to prevent the over-etching.

### Experimental setup

The confocal pinhole, diffraction grating, and CSIP were placed in a cryostat to maintain a temperature of ~ 4.2 K to operate the CSIP properly and remove any environmental noise owing to these optical components. The sample, probe, and Ge objective lens were kept at room temperature. The diffraction grating was designed to have more than 60% first-order diffraction efficiency and less than 8% second-order diffraction efficiency using the scalar theory of diffraction^20^. We machine the grating (material: oxygen-free Cu) by Ultra Precision Machine, ROBONANO (α-NMiA, FANUC corp.) with the cutting accuracy of 0.3%. The rotation angle of the grating was measured using a capacitance encoder (UNISOKU corp.).

The probe height was precisely controlled using a shear-force-mode AFM system and maintained at 5–10 nm above the sample surface during the near-field measurements^10,29^. The probe was attached to the quartz tuning fork and oscillated horizontally at the resonant frequency of the tuning fork (~ 31 kHz). The conductance of the tuning fork decreases when the probe-sample distance is less than 30 nm. The conductance of the tuning fork was monitored and kept constant using a feedback loop. The probe was also vertically vibrated at a frequency of 14 Hz to eliminate signals induced by background radiations. The change in the CSIP signals owing to the vertical vibration was regarded as the near-field signals. The near-field signals were obtained using a lock-in amplifier with a reference frequency of 14 Hz.

### Sample preparation

All samples have an Au strip with a thickness and width of 100 nm and 25 μm, respectively. The Au strip was fabricated by the thermal deposition method. The substrates are AlN or GaN crystals with thicknesses of 350 ± 15 μm on sapphire. All samples were cleaned with acetone and isopropyl alcohol (IPA) before the experiments.

### Supplementary Information

Supplementary Information.

## Data Availability

The data supporting the findings of this study are available from the corresponding author upon reasonable request.
