# Supplementary material for: Thermal near-field scattering characteristics for dielectric materials
Source: Sci Rep. 2023 Oct 16;13:17595. doi: 10.1038/s41598-023-44920-y (PMC10579336; doi:10.1038/s41598-023-44920-y)
Supplement: Supplementary file 1 — Supplementary Information. [file 41598_2023_44920_MOESM1_ESM.pdf]

Supplementary materials – Thermal Near-field Scattering Characteristics for Dielectric Materials–

Ryoko Sakuma,[1], Kuan-Ting Lin[2], and Yusuke Kajihara[1, 2, 3]

[1] *Department of Precision Engineering, The University of Tokyo, Bunkyo-ku, Tokyo, 113-8654, Japan*
[2] *Institute of Industrial Science, The University of Tokyo, Tokyo, Meguro-ku, 153-8505, Japan*
[3] *PRESTO, Japan Science and Technology Agency, Kawaguchi-shi, Saitama 332-0012 Japan*

Supplementary materials accompanying the manuscript, pertaining to the calculation of dispersion relation of GaN and AlN, decay lengths of evanescent waves, and all decay curves with uncertainty descriptions.

I. DISPERSION RELATION OF METAL AND DIELECTRIC MATERIALS

The dielectric constant of Au calculated in the paper was modelled by the Drude model with $\varepsilon_\infty = 1, \omega_p$ =72,800 cm^{-1}, and Γ =215[1]:

$$\varepsilon(\omega) = \varepsilon_\infty - \frac{\omega_p^2}{\omega^2 + i\Gamma\omega} \tag{S1}$$

where ε_∞, ω_p, and Γ are the dielectric constant at $z = \infty$, plasma frequency, and losses. The dielectric constant of dielectric materials can be modelled by the Loretz model, where ω_{LO} and ω_{TO} are the longitudinal and transverse optical frequencies[1]:

$$\varepsilon(\omega) = \varepsilon_\infty \left(1 + \frac{\omega_{LO}^2 - \omega_{TO}^2}{\omega_{TO}^2 - \omega^2 - i\Gamma\omega}\right) \tag{S2}$$

The ω_{LO} and ω_{TO} of AlN and GaN are given in Table S1. GaN and AlN are non-magnetic; therefore, only p-polariton—in which electric fields are parallel to the plain interface—exists as surface waves. The dispersion relation of the p-polariton waves at a AlN/vacuum and GaN/vacuum surface is given by Eq. (S3), where μ is the magnetic constant ($\mu = \mu_{\mathrm{AlN/GaN}} = \mu_{\mathrm{vacuum}}$). The calculated dispersion relations of AlN and GaN with real part of K are shown in Fig. S1. The dotted lines in Fig. S1 are the light line ($\omega = cK$). In general, surface waves are evanescent if the curve is below the light line. The Reststrahlen band is the energy band between the ω_{LO} and ω_{TO}. Using the dispersion relation of AlN and GaN, the electromagnetic LDOS can be calculated, as shown in Fig. 5(a). The maximum energy density can be found at the SPhP wavelength—that are 11.8 and 14.1 μm for AlN and GaN.

$$K^2 = \frac{\omega^2}{c^2}\mu\frac{\varepsilon_1(\omega)\varepsilon_2(\omega)}{\varepsilon_1(\omega) + \varepsilon_2(\omega)} \tag{S3}$$

Table S1. Parameters used to model dispersion relations[2,3].

	ω_{LO} [cm^{-1}]	ω_{TO} [cm^{-1}]	Γ
AlN	891	610	15.5
GaN	734	532	10

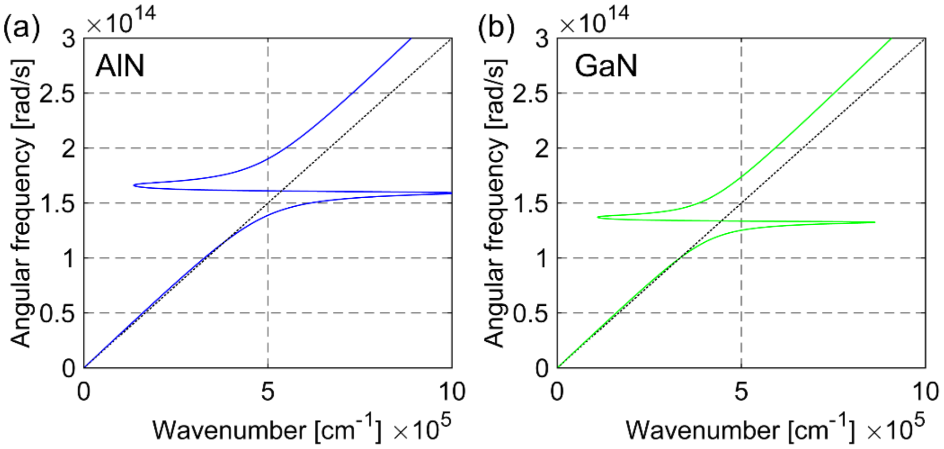

Figure S1. Dispersion relations (real parts) of (a) AlN and (b) GaN calculated using the Lorentz model.

II. POLARITON DECAY LENGTHS OF ALN, GAN, AND AU

The polariton decay length (δ) is the penetration length of light in medium and only reflects the information of the polariton component ($K{\sim}\omega/c$). The thermally excited evanescent waves contain various fluctuation modes of $\omega/c < K < \infty$; therefore, the polariton component is only a part of the thermally excited evanescent waves. Only in the Reststrahlen band, the influence of the polaritons become dominant. The polariton decay length is given by $\delta = 1/\mathrm{Im}(\gamma_{1,2})$[1]. The vertical component of a wavevector is denoted by $\gamma_{1,2}^2 = \varepsilon_{1,2}\mu_{1,2}k_0^2 - K^2$ (1 = vacuum, 2 = substrate material). Figure S2 shows the polariton decay length of GaN (green), AlN (blue), and decay length of higher fluctuation modes—in which surface plasmon polaritons do not emerge—of Au (black). The solid and dashed lines are the decay lengths in a vacuum and substrate material, respectively. If the SPhP wavelength is far enough from the detection wavelength—for example, Au in the infrared range—the decay length in a vacuum and substrate are ~10 nm and ~100 μm. It explains well the large energy losses in a vacuum. If the SPhP wavelength is within the detection wavelength range, the local minimum of the decay length in both the vacuum and material are at the SPhP wavelength. The decay length in a vacuum is ~1 μm at the SPhP wavelength.

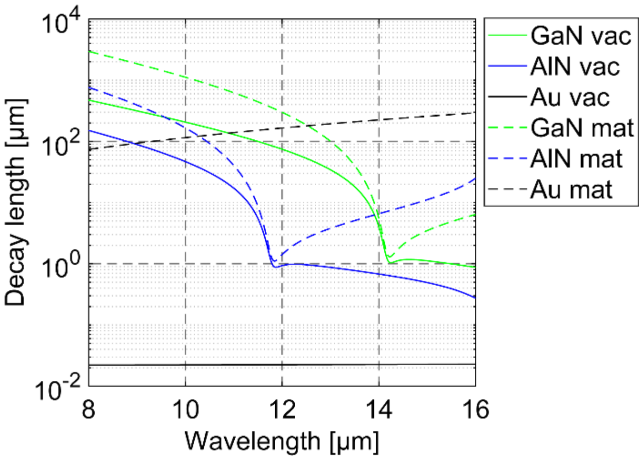

Figure S2. Decay length of the polariton components at the AlN/vacuum (blue) and GaN/vacuum (black) interface and decay length of higher fluctuation modes at the Au/vacuum interface (black). Dashed and solid lines show the decay length in a vacuum and substrate material, respectively.

III. PASSIVELY OBTAINED NEAR-FIELD DECAY CURVES

The spectroscopic measurements performed with passive LWIR spectroscopic s-SNOM is the detection of the first-order diffraction light. Because the wavelength resolution is ~150 nm whereas the spectral width of the CSIP is 1-2 μm[5], the intensity of the spectroscopic signal is an order of magnitude smaller than that of the zeroth-order diffraction waves. As shown in Fig. 5(b-f), the SNR of the spectroscopic signals on AlN and GaN were potentially large. The SNRs of each spectroscopic measurement of AlN and GaN are shown in Table S2 and S3, respectively. In the Reststrahlen band, relatively high SNRs were obtained near the polariton wavelengths: 11.8 μm for AlN and 14.1 μm for GaN. Figures S3(a-m) show average values of near-field signal intensity at each sampling point with shaded areas showing the standard deviations. Signal intensities were normalized to that of Au which was measured with the same probe.

Table S2. SNR of decay curves of spectroscopic measurements (AlN)

Wavelength [μm]	10.5	11.0	11.8	12.0	14.2	14.5	14.8
SNR [dB]	-1.01	-2.54	1.78	-3.41	3.94	4.45	4.91

Table S3. SNR of decay curves of spectroscopic measurements (GaN)

Wavelength [μm]	10.5	11.0	11.5	14.0	14.1	14.5
SNR [dB]	4.48	6.49	5.45	3.61	5.74	5.19

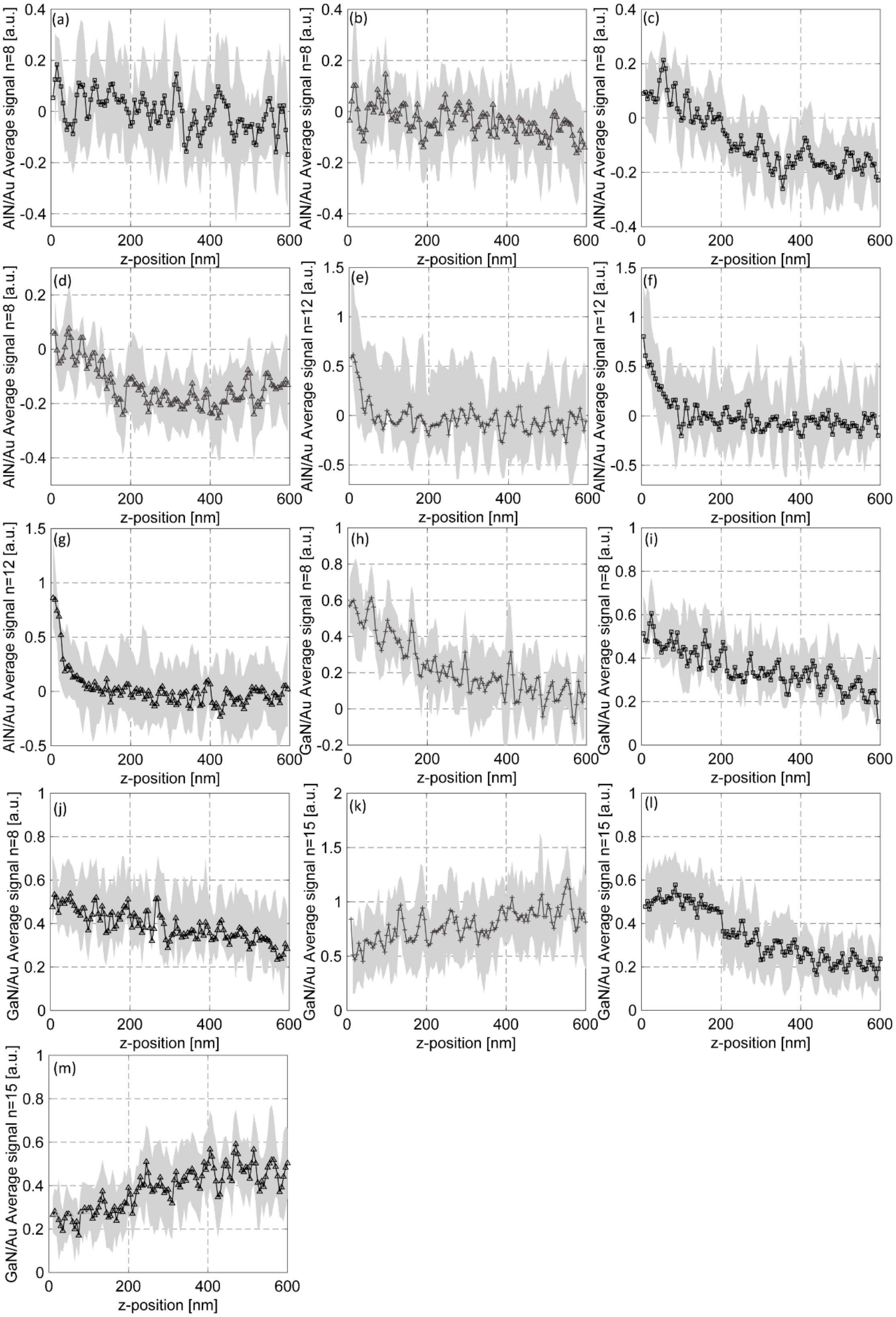

Figure S3. Passively obtained near-field decay curves on AlN and GaN with shaded area displaying uncertainties. (a-g) Decay curves on AlN at (a) 10.5, (b) 11.0, (c), 11.8, (d) 12.0, (e) 14.2, (f) 14.5, and (g) 14.8 μm. (h-m) Decay curves on GaN at (h) 10.5, (i) 11.0, (j) 11.5, (k) 14.0, (l) 14.1, and (m) 14.5 μm.

REFERENCE

1. Joulain, K., Mulet, J. P., Marquier, F., Carminati, R. & Greffet, J. J. Surface electromagnetic waves thermally excited: Radiative heat transfer, coherence properties and Casimir forces revisited in the near field. *Surface Science Reports* vol. 57 59–112 (2005).
2. Ng, S. S., Hassan, Z. & Abu Hassan, H. Experimental and theoretical studies of surface phonon polariton of AlN thin film. *Appl. Phys. Lett.* **90**, 081902 (2007).
3. Ng, S. S., Yoon, T. L., Hassan, Z. & Abu Hassan, H. Surface and interface phonon polaritons of wurtzite GaN thin film grown on 6H-SiC substrate. *Appl. Phys. Lett.* **94**, 92–95 (2009).
4. Joulain, K., Carminati, R., Mulet, J. P. & Greffet, J. J. Definition and measurement of the local density of electromagnetic states close to an interface. *Phys. Rev. B - Condens. Matter Mater. Phys.* **68**, 245405 (2003).
5. Sakuma, R., Lin, K.-T., Kim, S., Kimura, F. & Kajihara, Y. Passive near-field imaging via grating-based spectroscopy. *Rev. Sci. Instrum.* **93**, 013704 (2022).
